# Copeptin in the diagnosis and management of renal tubular disorders

**DOI:** 10.1007/s00467-025-06941-9

**Published:** 2025-09-09

**Authors:** Leire Madariaga, Angela Ferrulli, Marta García-Alonso

**Affiliations:** 1 Pediatric Nephrology Department, CIBERDEM/CIBERER/Endo-ERN, Biobizkaia Health Research Institute, University of the Basque Country, Cruces University Hospital, Plaza de Cruces S/N, Barakaldo, 48903 Spain; 2 Genomics and Proteomics Laboratory, Istituti Clinici Scientifici Maugeri IRCCS, Bari, Italy; 3 Pediatric Nephrology Department, Biobizkaia Health Research Institute, Cruces University Hospital, Barakaldo, Spain

**Keywords:** Copeptin, Polyuria-polydipsia syndrome, Renal tubular disorders, Biomarker

## Abstract

Copeptin, a stable glycopeptide derived from the precursor of arginine vasopressin (AVP), has emerged as a valuable surrogate biomarker for AVP due to its stability and ease of measurement. This narrative review explores the physiological role of copeptin, its utility as a diagnostic and prognostic biomarker in different kidney diseases, and its clinical relevance in renal tubular disorders. The clinical application of copeptin as a diagnostic biomarker is best established in the differential diagnosis of polyuria-polydipsia syndrome (PPS), distinguishing nephrogenic diabetes insipidus (NDI) from central diabetes insipidus (CDI) and primary polydipsia (PP). Baseline and stimulated copeptin levels demonstrate high diagnostic accuracy, although methodological and population-specific limitations exist, especially in pediatrics. Copeptin has also proved to be a marker of disease severity in a wide range of acute pathologies. In chronic kidney disease (CKD), it correlates negatively with kidney function, and it has been shown to be a marker of kidney function decline in kidney transplant patients and in autosomal dominant polycystic kidney disease (ADPKD). Regarding renal tubular disorders, CKD has increasingly been recognized in these patients, potentially driven by persistent volume depletion and activation of the renin–angiotensin–aldosterone system. Copeptin may offer an objective assessment of volume status and disease severity, particularly in infants and young children. However, further studies are needed to define standardized reference values, clarify its mechanistic role, and validate its prognostic utility in tubulopathies. Copeptin holds potential as both a diagnostic and prognostic biomarker in renal tubular disease, with implications for clinical practice and patient management.

**Graphical abstract:**

A higher resolution version of the Graphical abstract is available as Supplementary information. 
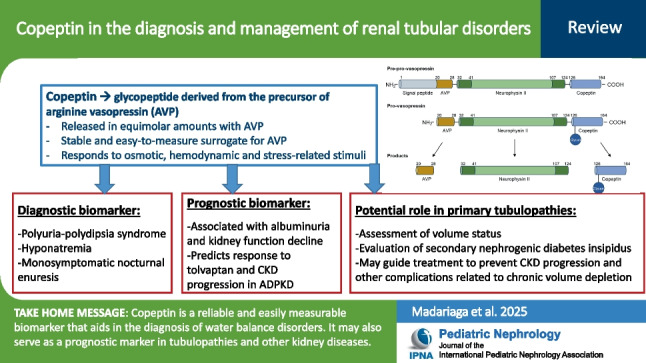

**Supplementary information:**

The online version contains supplementary material available at 10.1007/s00467-025-06941-9.

## Introduction

### Characteristics of copeptin

**Fig. 1:**
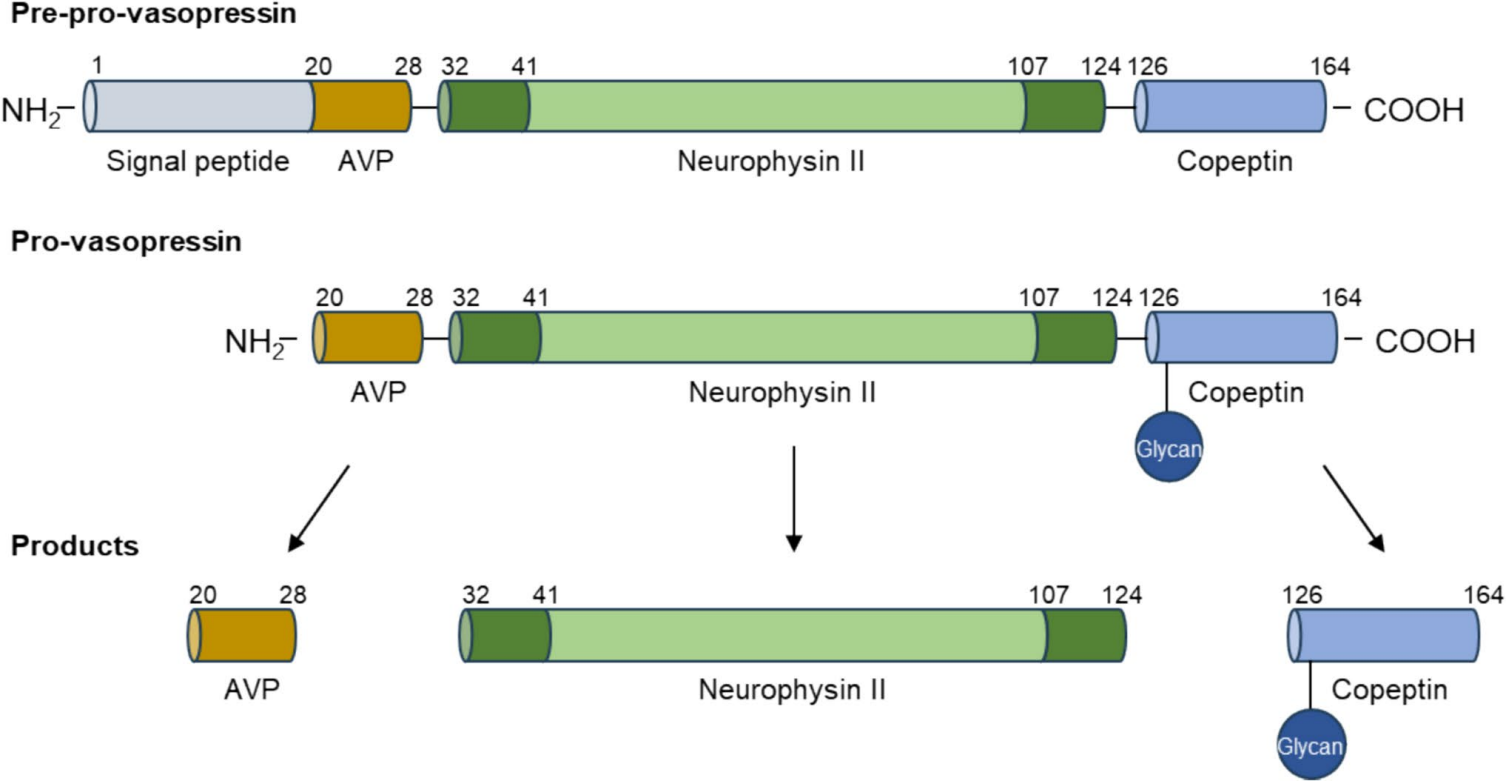
Schematic structure of pre-pro-vasopressin. The 164-amino acid precursor consists of a signal peptide, AVP, neurophysin II, and copeptin. Enzymatic cleavage of the precursor hormone generates three peptides: AVP, neurophysin II, and copeptin

Copeptin is a leucine-rich glycosylated peptide, consisting of 39 amino acids. It derives from the C-terminal portion of the 164 amino acid precursor protein pre-pro-vasopressin together with arginine vasopressin (AVP) and neurophysin II synthesized in the magnocellular neurons of the hypothalamus [1]. The cleavage of the signal peptide from pre-pro-vasopressin results in the formation of pro-vasopressin. As the latter is transported along the axon through the pituitary stalk to the neurohypophysis, it is processed into the three distinct components: AVP, neurophysin II, and copeptin (Fig. 1). Copeptin is released into the circulation together with AVP in equimolar amounts, thus reflecting the concentration of AVP in both plasma and human serum [2].

### Copeptin in physiological processes and renal tubular homeostasis

First described in 1972 by Holwerda [3], copeptin has gained considerable interest in recent decades for its clinical relevance as a diagnostic and prognostic biomarker in various conditions, including diabetes insipidus (DI), chronic kidney disease (CKD), sepsis, and cardiovascular diseases [4].

Nevertheless, the physiological role of copeptin is not fully understood. Initially, copeptin was believed to stimulate prolactin release, but this hypothesis yielded inconclusive results [5]. More recently, copeptin has been proposed to facilitate the folding of the vasopressin precursor, through the calnexin/calreticulin system [6], which monitors protein folding and interacts with glycosylated proteins, supporting the formation of the active AVP. Therefore, a loss of copeptin could contribute to the pathogenesis of some forms of central diabetes insipidus (CDI) [7]. Copeptin is rapidly eliminated from the bloodstream, likely by tissue-bound proteases or clearance by the kidneys or liver. This rapid elimination does not occur ex vivo. Even though no specific receptors or elimination mechanisms have been identified, copeptin decay kinetics is different from that of AVP, suggesting that this peptide is not only a side product of AVP synthesis but has some specific role [8].

Copeptin is released into the bloodstream in response to osmotic and hemodynamic changes or nonspecific stress, in stoichiometric concentrations to AVP, thereby reflecting its secretion dynamics [9]. Copeptin levels are highly sensitive to hydration status, increasing with osmotic stimulation (e.g., water deprivation or hypertonic saline) and decreasing with hypotonic fluid intake. This was demonstrated in a study involving 20 healthy volunteers, where mean copeptin concentrations decreased from 3.3 pM (range, 1.1–36.4) at baseline to 2.0 pM (range, 0.9–10.4) following water loading and increased to 13.6 pM (range, 3.7–43.3) after hypertonic saline infusion. These changes mirrored AVP levels and correlated closely with plasma osmolality [10].

Physiologically, AVP is secreted from the posterior pituitary in response to elevated plasma osmolality or decreased blood pressure and volume. In renal principal cells, AVP binds to V2 receptors, activating the Gs–adenylyl cyclase–cAMP–PKA signaling pathway, leading to phosphorylation and apical membrane insertion of aquaporin-2 (AQP2), thereby enhancing water reabsorption [11].

Additionally, AVP exerts vasoconstrictive effects via V1a receptor-mediated activation of phospholipase C-β in vascular smooth muscle [12] and modulates the stress response through V1b receptor activation in the anterior pituitary and adrenal glands, stimulating ACTH and catecholamine secretion [13]. In this regard, copeptin also represents a sensitive marker of acute physical and psychological stress, significantly increasing not only in response to various acute diseases, such as myocardial infarction, sepsis, and stroke, but also following psychological stressors [14].

Considering the essential role of vasopressin in water balance, the assessment of endogenous plasma AVP is crucial in the diagnosis and management of water and electrolyte balance disorders, including renal tubular diseases. However, direct measurements of plasma AVP can be challenging, Due to its short half-life in vivo of approximately 24 min [15]. Moreover, because AVP binds to V1 receptor expressed on platelets, incomplete removal of platelets from plasma or long-term storage of unprocessed samples can lead to inaccurate estimates of free plasma AVP [16]. In addition, the small size of vasopressin impedes the proper binding of two antibodies necessary in sensitive sandwich immunoassays [17].

### Measurement of copeptin in clinical setting and its limitations

Copeptin has been successfully used as a valuable surrogate biomarker for vasopressin release, offering several advantages over AVP in clinical settings. Copeptin measurements only require small volumes of serum or plasma (50 µL) and do not necessitate complex pre-analytical procedures. Unlike AVP, copeptin shows greater stability ex vivo both in plasma and in serum [18]. It is reported that copeptin shows < 20% loss of recovery for at least 7 days at room temperature and for 14 days at 4 °C, making sample handling simpler than for AVP measurements [19]. Moreover, copeptin has been shown to have better correlation with plasma osmolality than AVP [10], suggesting that measuring copeptin may be even more reliable than AVP. In clinical routine, copeptin can be measured using two commercially available CE-certified assays: the original manual sandwich immunoluminometric assay (LIA) and its automated immunofluorescent version on the KRYPTOR platform, both offering high-standard technical performance [20]. Other commercially available assays are mainly represented by the ELISA assays, which are not CE-certified and cannot be used for diagnostic purposes.

Copeptin levels in healthy subjects are quite variable. In a study conducted in 359 healthy individuals (153 men and 206 women), Morgenthaler et al. found that median copeptin values were 4.2 pmol/L, ranging from 1 to 13.8 pmol/L. They observed no significant differences in median copeptin concentration among the age groups, indicating the absence of a correlation with age [19]. Notably, median copeptin levels are higher in males than in females. It has also been shown that copeptin levels increase following physical exercise and are influenced by factors such as fasting and water load [19]. Additionally, corticosteroids have been shown to inhibit copeptin secretion [21].

## The role of copeptin as a diagnostic biomarker in disorders associated with polyuria or electrolyte imbalances

Copeptin levels have been associated with a wide range of pathologies, in most cases as a marker of disease severity. These include diabetes mellitus [22], sepsis [23], cardiovascular diseases [24], respiratory diseases [25], and traumatic brain injury [26], among others. However, the most robust and clinically validated application of copeptin measurement is as a diagnostic tool in the differential diagnosis of the polyuria-polydipsia syndrome (PPS) [27].

### Polyuria-polydipsia syndrome

The differential diagnosis of the PPS with low urinary osmolality mainly focuses on three entities: central diabetes insipidus (CDI; also known as arginine vasopressin deficiency or AVP-D), nephrogenic diabetes insipidus (NDI, also known as arginine vasopressin resistance or AVP-R), and primary polydipsia (PP). Traditionally, the water deprivation test (WDT) with or without desmopressin administration (synthetic AVP analogue) has been considered the gold standard diagnostic method. However, its clinical utility is limited by its relatively low diagnostic accuracy (≈ 70%), and by the fact that it is also an uncomfortable, time-consuming, and costly procedure [9, 28]. Considering these limitations, the use of copeptin as a diagnostic tool has been extensively evaluated (Table 1).

**Table 1:** Main research studies involving the use of copeptin as a diagnostic or prognostic biomarker in kidney diseases

First author and year	Population, follow-up time (if applicable)	Disease	Prognosis versus diagnosis biomarker	Main finding	Reference
Dawman 2024	110 children, 0–14 years (60 cases and 50 controls). Follow-up time: 1 year	CKD 2–4	Prognosis	No correlation between change in eGFR and copeptin	[29]
Tasevska 2016	3186 adults, mean follow-up time: 16.6 years	None	Prognosis	Copeptin is an independent predictor of eGFR decline and risk of new-onset CKD	[30]
El Boustany 2018	13,597 adults, median follow-up time: 12.1 years	CKD	Prognosis	High copeptin levels are associated with development and progression of CKD	[31]
Meijer 2009	548 adults, median follow-up time: 3.2 years	Kidney transplant	Prognosis	Elevated copeptin level is associated with accelerated renal function decline	[32]
Gansevoort 2019	1280 adults (18–50 years) treated with tolvaptan. Follow-up time: 3 years	ADPKD	Prognosis	Higher increases of copeptin in tolvaptan-treated subjects present a better disease outcome	[33]
Boertien 2012	241 adult patients, mean follow-up time: 8.5 years	ADPKD	Prognosis	High copeptin levels are associated independently with disease progression	[34]
Fathi 2023	87 children (58 cases, 29 controls)	Primary MNE	Prognosis	Copeptin levels are significantly lower in children with primary MNE compared to healthy controls	[35]
Sailer 2023	28 children (5–16 years)	MNE treated with desmopressin	Prognosis	Copeptin evening to morning ratio is the best predictor for treatment response	[36]
Fenske 2018	156 adults	PPS	Diagnosis	Hypertonic saline-stimulated copeptin has significantly greater diagnostic accuracy than the WDT	[37]
Trimpou 2024	88 adults	PPS	Diagnosis	Copeptin > 4.0 pmol/L after WDT rules out CDI. Copeptin ≥ 21 pmol/L at baseline is diagnostic for NDI	[38]
Bonnet 2022	278 children, 2 months to 18 years (40 cases, 238 controls)	PPS	Diagnosis	Basal copeptin < 3.53 pmol/L is diagnostic for CDI	[39]
Timper 2015	55 adults	PPS	Diagnosis	Copeptin > 21.4 pmol/L is diagnostic for NDI. Stimulated copeptin > 4.9 pmol/L differentiates CDI from PP	[40]

*ADPKD* autosomal dominant polycystic kidney disease, *CDI* central diabetes insipidus, *CKD* chronic kidney disease, *eGFR* estimated glomerular filtration rate, *MNE* monosymptomatic nocturnal enuresis, *NDI* nephrogenic diabetes insipidus, *PP* primary polydipsia, *PPS *polyuria-polydipsia syndrome, *WDT* water deprivation test

Among the three entities that mainly account for PPS, the role of copeptin in the diagnosis of NDI is the most supported in the literature. NDI is characterized by a deficient response of the V2R to the action of AVP. It can be primary, due to variants in the gene that encodes the V2R (*AVPR2*) or the water channel AQP2 (*AQP2*), or secondary to medications, electrolyte imbalances, or other pathologies [41]. Secondary NDI due to chronic lithium treatment is the most common cause worldwide [42].

Several studies have shown that elevated baseline copeptin concentrations allow for the diagnosis of NDI in patients with PPS with very high sensitivity and specificity (both as high as 100% in some series [40]), without the need for prior WDT or other stimulation techniques. Proposed cut-off values include > 21.4 pmol/L [40] or > 20 pmol/L [38] in adults, and > 20 pmol/L [43] or > 30 pmol/L [39] in the pediatric population. However, these data should be interpreted with caution, as the published series include small sample sizes of patients with NDI, especially in children. Furthermore, other studies have reported significantly elevated baseline copeptin concentrations in up to 9% of healthy children, most likely related to stress induced by venepuncture, which could lead to diagnostic errors [44]. Larger studies comparing patients with NDI to healthy controls are needed, particularly in pediatric populations, who may experience higher levels of stress during venepuncture. Similar considerations apply to adults who may be prone to distress or suffer from anxiety-related disorders. Additionally, Vergier et al. described a pediatric case of primary NDI in which copeptin levels normalized after rehydration, suggesting its potential usefulness as a marker of therapeutic response in this context [45].

The interpretation of copeptin in the differential diagnosis between CDI and PP remains more controversial, Due to the heterogeneity of the published results and the lower consistency of the available evidence. In 2011, Fenske et al. proposed basal copeptin cut-off values of < 2.6 pmol/L for CDI and > 20 pmol/L for NDI, with 100% specificity, although there was significant overlap between CDI and PP in the intermediate values [28]. This finding was later confirmed by another group, who observed that 61% of patients with PP had a lower basal copeptin than the highest value in the CDI group (3.3 pmol/L). Although the overlap decreased, it remained significant at the end of the WDT. However, values > 4 pmol/L after 8 h of WDT ruled out CDI, potentially reducing the number of patients who would require prolonged water deprivation or other tests to reach a diagnosis [38].

To increase diagnostic accuracy, different techniques for stimulating copeptin secretion have been evaluated, traditionally using hypertonic saline [46]. This technique provides greater diagnostic accuracy compared to WDT alone (96.5% vs. 76.6%). Proposed cut-off values for copeptin stimulated by hypertonic saline include > 4.9 pmol/L to differentiate PP from CDI, with 94% sensitivity and 96% specificity [40], or more recently, < 2.7 pmol/L for complete CDI, 2.7–4.9 pmol/L for partial CDI, and > 4.9 pmol/L for PP (with 93.2% sensitivity and 100% specificity for values < 4.9 pmol/L to differentiate CDI from PP) [37].

Nevertheless, the use of hypertonic saline has some limitations, especially in the pediatric population, including adverse effects, the need for good vascular access for frequent monitoring, hospital stays, and a lack of safety data in patient groups [47]. Therefore, other non-osmotic stimulation techniques, such as arginine, have been evaluated. Proposed cut-off values for this technique include < 2.4 pmol/L for complete CDI, 2.4–3.8 pmol/L for partial CDI, and > 3.8 pmol/L for PP, with 93% sensitivity, 92% specificity, and 93% diagnostic accuracy for values < 3.8 pmol/L to differentiate CDI from PP [48]. However, a later study failed to demonstrate the non-inferiority of arginine stimulation compared to hypertonic saline stimulation [47], reporting a diagnostic accuracy of 74.4% with arginine versus 95.6% with hypertonic saline, using the previously proposed cut-off values.

In the pediatric population, published data are more limited, and cut-off values similar to those described in adults have been suggested. For example, after 6–10 h of water deprivation, a copeptin concentration of < 2.2 pmol/L has been proposed for complete CDI, 5–20 pmol/L for PP, and > 20 pmol/L for NDI, with overlap between CDI and PP in the 2.2–5 pmol/L range (with a 75% probability of CDI for values of 2.2–3.5 pmol/L, compared to 25% for values of 3.5–5 pmol/L) [39]. Subsequently, other groups have proposed similar values for both basal copeptin [39] and arginine stimulation [49].

A recent systematic review and meta-analysis on the diagnostic utility of copeptin in pediatric patients with PPS [50] reported a sensitivity of 95–100%, specificity of 85–100%, and diagnostic accuracy ranging from good to excellent in the majority of studies (combined AUC, area under the curve—0.972).

In a recently published international consensus on the diagnosis and management of congenital NDI, it is stated that elevated copeptin levels (≥ 21.4 pmol/l) are suggestive of NDI in adults, although this has not been validated in children. This may be particularly important in settings where genetic testing is unavailable or inconclusive [41].

### Hyponatremia

Hyponatremia is the most common electrolyte disturbance in hospitalized patients, with SIADH (syndrome of inappropriate antidiuretic hormone secretion) being one of the most frequent causes in both children and adults [9, 51]. Patients with SIADH may present with euvolemic hyponatremia and a decreased ability to dilute urine. In this context, NSIAD (nephrogenic syndrome of inappropriate antidiuresis) is an extremely rare inherited disorder caused by a gain-of-function variant in the gene encoding V2R (*AVPR2*) and is considered the mirror image of NDI. It results in hyponatremia due to increased free water reabsorption. Clinical suspicion should arise in cases that resemble SIADH but have low or undetectable AVP and/or copeptin levels, particularly when there is a positive family history. As an X-linked disorder, NSIAD affects males, while female carriers are asymptomatic or present mild symptoms. This is especially relevant in early childhood when no other cause of SIADH is evident [52].

In patients with hyponatremia, it has been observed that the copeptin/urinary sodium ratio < 30 pmol/mmol is more accurate than the isolated copeptin concentration [53] or isolated urinary sodium and serum uric acid [54] in differentiating SIADH from other causes of hyponatremia. Additionally, evaluating copeptin dynamics could help distinguish between the different subtypes of SIADH, which may be useful in the differential diagnosis of its etiology [9].

Although in hyponatremic patients the lowest and highest copeptin concentrations have been described in PP and hypovolemic hyponatremia, respectively [53], the differential diagnosis of other causes of hyponatremia (including SIADH) using copeptin remains challenging due to the overlap between clinical entities.

### Monosymptomatic nocturnal enuresis

Monosymptomatic nocturnal enuresis (MNE) is a common issue in pediatrics, with a prevalence of approximately 10% at age 7 [55]. One of the medications used is desmopressin, which, although generally safe, is not without risks [56]. For this reason, the role of copeptin has been evaluated to identify patients who are most likely to benefit from this treatment. In patients with MNE, lower copeptin concentrations have been observed compared to healthy children [35, 57], and it has been suggested that the ratio of evening/morning basal copeptin is a good predictor of desmopressin responsiveness (AUC 0.70, with a cutoff of 1.34) [36].

## The role of copeptin as a prognostic biomarker in kidney diseases

In the general population, copeptin has been shown to correlate negatively with kidney function [31]. However, the mechanism underlying this association is not clearly defined and is likely multifactorial, rather than solely related to volume depletion. Copeptin is independently associated with albuminuria, which is a known marker of kidney disease progression [58]. In addition, it has been shown to correlate with kidney function decline in transplant patients [32]. Copeptin levels in the general population also correlate with lifestyle and diet-related factors which are traditionally linked to CKD, such as smoking, alcohol use, and sodium intake [59]. In autosomal dominant polycystic kidney disease (ADPKD), which is the most common inherited kidney disorder associated with CKD in the adult population, the stimulation of V2R receptors in the collecting duct by AVP stimulates the generation of cAMP, and this in turn increases the volume of kidney cysts and the loss of normal kidney parenchyma. In these cases, higher copeptin concentration has been associated with kidney function decline during follow-up in some studies [34]. Moreover, in patients with ADPKD treated with the V2R antagonist tolvaptan, a larger increase in copeptin levels after initiation of treatment has been associated with a better disease outcome [33].

However, in all these conditions, it is not clear whether copeptin is a marker of kidney disease because it reflects the vascular effects of AVP via the V1 receptors or its fluid and osmotic homeostatic effects via the kidney V2 receptors (Table 1). Moreover, copeptin could also play a pathogenic role in the pathophysiology of these diseases, rather than merely serving as a biomarker of disease progression.

In kidney disorders associated with polyuria due to a decrease in the maximum urinary concentration capacity, it is often difficult to evaluate the severity of volume depletion in clinical settings in an objective way. This is especially important in infants, who cannot voluntarily satisfy their thirst and may suffer from chronic volume depletion. In these cases, copeptin as a surrogate of AVP could act as an objective marker of volume depletion.

## The potential role of copeptin in the management of primary tubulopathies

Although the role of copeptin has been evaluated in relation to the diagnosis and/or prognosis of a wide range of diseases, to date, no studies have been published assessing its role in the clinical diagnostic work-up of kidney tubulopathies other than NDI. Measuring copeptin as a good biochemical surrogate of AVP could be particularly useful in assessing volume status and serving as a prognostic marker in patients with polyuric tubulopathies such as Dent syndrome, Bartter syndrome, Gitelman syndrome, distal renal tubular acidosis, renal Fanconi syndrome, or cystinosis. Secondary NDI has been proposed in many primary tubulopathies and can be difficult to assess, especially in infants. In addition, volume depletion and secondary stimulation of the renin–angiotensin–aldosterone system (RAAS) may act as a prognosis factor for the development of CKD over time in these patients.

### Secondary NDI in primary tubulopathies

Polyuria with low urinary concentrating ability has been shown in some primary tubulopathies other than NDI, namely, Bartter syndrome, renal Fanconi syndrome, dRTA, and familial hypomagnesemia with hypercalciuria and nephrocalcinosis. However, secondary NDI as the cause of this polyuria, with low urinary concentrating ability and limited response to AVP, has only been proved in some [60, 61]. Bartter syndrome type 1 and 2, secondary to biallelic pathogenic variants in *SCL12A1* and *KCNJ1* genes encoding the Na–K-2Cl NKCC2 cotransporter and the ROMK potassium channel, respectively, typically present with a severe and early polyhydramnios and a sustained polyuria during childhood. Resistance to AVP effect, indicating secondary NDI, has been demonstrated in some patients with marked polyuria, hyposthenuria, and a lack of response to AVP along with a tendency toward hypernatremia with regular salt supplementation for Bartter syndrome [60, 61]. Evaluating these patients for secondary NDI is recommended, as salt supplementation may worsen polyuria and increase the risk of hypernatremic dehydration [62]. However, this is not consistently reported, and in our experience, some patients maintain isosmotic urines or even higher urine than plasma osmolality with fasting stimuli. In salt-losing tubulopathies affecting the loop of Henle, the reabsorption of sodium and chloride is impaired, and this activates the macula densa, increasing the expression of the enzyme cyclooxygenase-2 (COX-2), which leads to the release of prostaglandin E2 (PGE2). PGE2 regulates renal hemodynamics stimulating the vasodilation of the afferent arteriole. PGE2 also directly inhibits the sodium reabsorption in the collecting duct [63] and acts on the collecting duct enhancing AQP2 endocytotic uptake from the apical plasma membrane, thus inhibiting the action of AVP [64]. Therefore, the hypersecretion of PGE2 in Bartter syndrome, by inhibiting AVP action, exacerbates polyuria, resulting in secondary NDI. In addition, PGE2 may also regulate AVP synthesis in the hypothalamus [65].

Moreover, it has also been proposed that in Bartter syndrome—similar to the postoperative period of patients who have undergone pyeloureteral junction obstruction surgery and subsequently present with polyuria—the high urinary flow reaching the collecting duct alters the expression of AQP2 channels at the apical membrane, contributing further to the mechanism of secondary NDI [66].

Polyuria associated with secondary NDI has also been described in proximal tubulopathies. In nephropathic cystinosis, polyuria/polydipsia is a typical feature of the disease, and it is in part due to decreased proximal solute and fluid reabsorption. However, secondary NDI has also been described in some patients with nephropathic cystinosis who present with low urinary osmolality (below plasma osmolality) and no response to AVP [60].

In all these settings, copeptin may help in the assessment of polyuric primary tubulopathies in which water losses due to secondary NDI are increased. These patients may require more intensive hydration and closer monitoring during acute conditions that could exacerbate dehydration and further impair kidney function.

### Volume depletion in primary tubulopathies

In recent years, CKD has increasingly been recognized as a common complication of several primary tubulopathies previously considered benign. Patients with Bartter syndrome type 3 may develop CKD over time in 25% of cases [67]. Adult patients with NDI were shown to have CKD grade 2 or more in 48% of cases in a wide international cohort [68]. Others, such as nephropathic cystinosis, almost universally develop kidney failure in the first decades of life.

Different factors may be involved in this long-term complication, namely, frequent decompensations with acute kidney failure, nephrocalcinosis, the use of indomethacin and other non-steroidal anti-inflammatory drugs (NSAID), persistent hypokalemia, and persistent volume depletion. This last factor is relevant as a risk factor for the development of CKD over time as it is one of the main stimuli for the activation of the RAAS.

The importance of volume depletion and consequent RAAS activation as a relevant factor for CKD over time has been reported in a comparative study with patients with Gitelman and Bartter syndrome [69]. Patients with Gitelman syndrome presented with lower mean potassium levels than patients with Bartter syndrome, but the latter had higher aldosterone levels and lower kidney function, concluding that water losses and consequent secondary hyperaldosteronism would probably be more implicated in the development of CKD than hypokalemia.

### Measurement of copeptin in kidney tubulopathies

Copeptin has been evaluated as a diagnostic tool in primary tubulopathies associating polyuria, namely, NDI, but not as a prognosis biomarker in these disorders. Random measurements of copeptin in clinical settings in our patients with different primary tubulopathies have shown clearly higher levels in those with a known worse kidney function prognosis, such as cystinosis or Bartter 4 syndrome. These were even higher than in patients with treated primary NDI. However, copeptin levels vary with water intake, and this factor is difficult to control especially in infants and young children with primary tubulopathies associated with severe polyuria.

It is not clear whether copeptin level in these settings increases in a similar way to aldosterone levels, which have traditionally been used together with other clinical parameters to assess volume status in polyuric patients with tubulopathies. RAAS activation has been linked to progressive CKD because of the profibrotic effects of aldosterone and to worse quality of life in these patients [70]. However, volume depletion itself is an important factor in the short term that impedes normal growth and development in children with primary tubulopathies [71]. In conclusion, due to its stability and easy measurement in clinical settings, copeptin as a surrogate of AVP could potentially be an additional biomarker of volume status in these patients and help in clinical assessment and treatment.

## Conclusions

Copeptin is a valuable surrogate of AVP and has been shown to be a good diagnostic tool in different diseases associated with polydipsia-polyuria syndrome. In addition, it may be valuable as a prognostic marker in several kidney diseases. However, its levels should be interpreted with caution, as they can be influenced by various pathophysiological factors, and definitive reference values are not yet established. Current evidence suggests that the greatest diagnostic utility of copeptin lies in the evaluation of NDI, particularly in the diagnostic work-up of pediatric patients with PPS given the high prevalence of genetic etiologies in this population. This is especially relevant in low-resource countries, where genetic testing may not be available. Further studies are needed to evaluate the role of copeptin as a biochemical marker of volume status in the management of tubulopathies that associate polyuria.

## Supplementary information

Below is the link to the electronic supplementary material.

Graphical abstract (PPTX 140 KB)

## Declarations

### Disclaimer

The funders had no role in the study design, data collection and analysis, decision to publish, or preparation of the manuscript.
