# Supplementary material for: Copeptin in the diagnosis and management of renal tubular disorders
Source: Pediatr Nephrol. 2025 Sep 9;41(5):1249–58. doi: 10.1007/s00467-025-06941-9 (PMC13008999; doi:10.1007/s00467-025-06941-9)
Supplement: Supplementary file 1 — Graphical abstract (PPTX 140 KB) [file 467_2025_6941_MOESM1_ESM.pptx]

## Slide 1
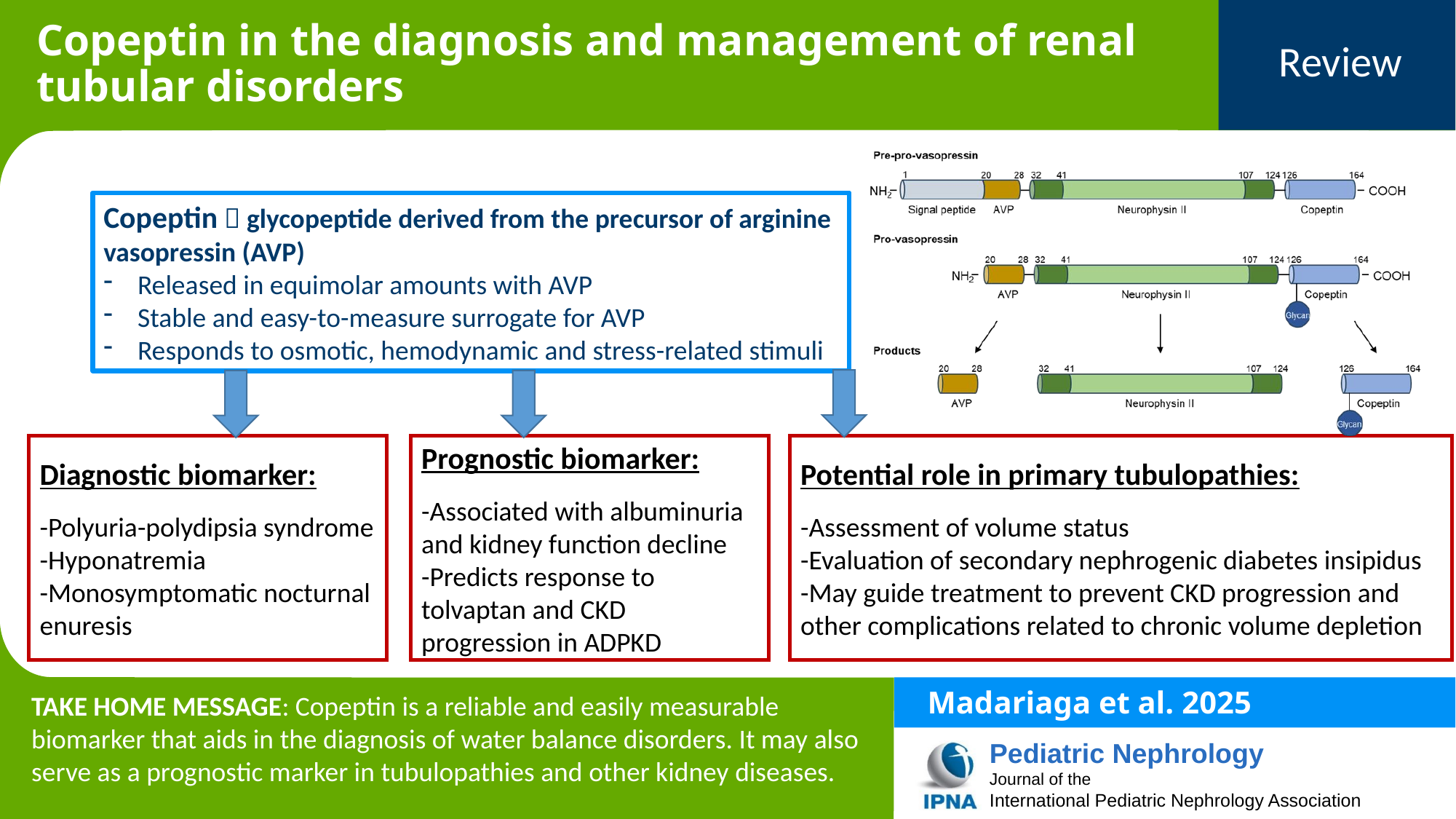

Copeptin in the diagnosis and management of renal tubular disorders
Copeptin  glycopeptide derived from the precursor of arginine vasopressin (AVP)
Released in equimolar amounts with AVP
Stable and easy-to-measure surrogate for AVP
Responds to osmotic, hemodynamic and stress-related stimuli
Diagnostic biomarker:
-Polyuria-polydipsia syndrome
-Hyponatremia
-Monosymptomatic nocturnal enuresis
Prognostic biomarker:
-Associated with albuminuria and kidney function decline
-Predicts response to tolvaptan and CKD progression in ADPKD
Potential role in primary tubulopathies:
-Assessment of volume status
-Evaluation of secondary nephrogenic diabetes insipidus
-May guide treatment to prevent CKD progression and other complications related to chronic volume depletion
Madariaga et al. 2025
TAKE HOME MESSAGE: Copeptin is a reliable and easily measurable biomarker that aids in the diagnosis of water balance disorders. It may also serve as a prognostic marker in tubulopathies and other kidney diseases.
